# Hydrogel Cryopreservation System: An Effective Method for Cell Storage

**DOI:** 10.3390/ijms19113330

**Published:** 2018-10-25

**Authors:** Chaocan Zhang, Youliang Zhou, Li Zhang, Lili Wu, Yanjun Chen, Dong Xie, Wanyu Chen

**Affiliations:** School of Materials Science and Engineering, Wuhan University of Technology, Wuhan 430070, China; polymers@whut.edu.cn (C.Z.); zyl_2013@whut.edu.cn (Y.Z.); polymerzl@163.com (L.Z.); polym_wl@whut.edu.cn (L.W.); yanjunchen@whut.edu.cn (Y.C.); xdong@whut.edu.cn (D.X.)

**Keywords:** cryopreservation, hydrogel, cell storage, supramolecular gel

## Abstract

At present, living cells are widely used in cell transplantation and tissue engineering. Many efforts have been made aiming towards the use of a large number of living cells with high activity and integrated functionality. Currently, cryopreservation has become well-established and is effective for the long-term storage of cells. However, it is still a major challenge to inhibit cell damage, such as from solution injury, ice injury, recrystallization and osmotic injury during the thawing process, and the cytotoxicity of cryoprotectants. Hence, this review focused on different novel gel cryopreservation systems. Natural polymer hydrogel cryopreservation, the synthetic polymer hydrogel cryopreservation system and the supramolecular hydrogel cryopreservation system were presented, respectively. Due to the unique three-dimensional network structure of the hydrogel, these hydrogel cryopreservation systems have the advantages of excellent biocompatibility for natural polymer hydrogel cryopreservation systems, designability for synthetic polymer hydrogel cryopreservation systems, and versatility for supramolecular hydrogel cryopreservation systems. To some extent, the different hydrogel cryopreservation methods can confine ice crystal growth and decrease the change rates of osmotic shock in cell encapsulation systems. It is notable that the cryopreservation of complex cells and tissues is demanded in future clinical research and therapy, and depends on the linkage of different methods.

## 1. Introduction

At present, various living cells such as embryonic stem cells [1,2,3], germ cells [4,5], neural cells [6,7,8] and hepatocytes [9,10,11] have been in development in the fields of cell therapies, regenerative medicine, stem cell research [12], organ transplantation and the study of endangered species [13]. For these cell-based applications, it is particularly important that a sufficient number of cells with high activity and corresponding integrated functionality survive for the whole process. However, living cells, especially germ cells and embryonic stem cells, find it extremely difficult to survive in vitro. Fortunately, the biological activities of cells can be inhibited after the cells are cooled down to a definite temperature, and the functions of cells are also recovered after cells are thawed at physiological temperature [14]. Hence, the cryopreservation of cells has become a currently widespread method and is also used for the long-term storage of living cells and donor tissue.

The change of solution concentration, water state and osmotic equilibrium in cells can cause serious cell damage. Thus, the conventional methods, such as slow freezing (or conventional freezing) and ice-free cryopreservation (or vitrification) [15,16], are accepted to cryopreserve various cells types, tissues and organs [17]. During the slow freezing process, a programmed cooling process was stepped along with cell dehydration, which avoided the formation of ice crystals from the intracellular water. A low cryoprotectant agent (CPA) was added to prevent the concentration of electrolytes in cells. A cryoprotectant is distinguished from permeable cryoprotectants (PCs) and impermeable cryoprotectants (IPCs). PCs can produce a strong hydration to partly replace the intracellular water [18]. Correspondingly, IPCs are effective to reduce the amount of PCs and accelerate the glass transition process for reducing ice crystal formation [19]. When neural stem cell (NSC) spheres of different sizes and states were frozen in the slow freezing process [20], the cell viability and induced differentiation depended on the concentration of dimethyl sulfoxide (DMSO) and the diameter of NSC spheres, and a survival rate of 82.9% with normal multi-differentiation potentiality was obtained with optimal parameters. By the slow freezing method, the protective natures of cells were accomplished via the permeability of CPAs and cryopreservation. The factors, which referred to the diffusion effect of CPAs through cell membranes, such as the preserved state and the size of NSC spheres, would influence the cryopreservation effect. While ovarian tissue was slow frozen using sucrose as a cryoprotectant [21], the follicular viability increased to 1.9 ± 0.2 (number of positive follicles per 0.0625 mm^2^), and also obtained higher normal primordial follicles than the group without sucrose. Thus, the CPAs could significantly influence the cell cryopreservation in the slow freezing method. Vitrification is a modern cryopreservation technique based on an extremely rapid cooling rate of cells along with the process of the extreme concentration of CPAs in cell suspension [22], which can decrease the freezing temperature before sudden cooling [23]. During the vitrification process, cell culture media with an extreme concentration of CPAs passed a rapid transition from liquid to glass, which minimized the cell damage. Both methods have their drawbacks for cells in cryo-injury during the cryopreservation process, including ice injury [24,25] from the ice crystal formation of cooling process, osmotic injury [26] from the loading and unloading of cryoprotectant agents (CPAs), and the toxicity of CPAs. Limited by the permeability and concentration of CPAs, both cryopreservation methods may cause a loss of cell viability and function, due to the toxicity of CPAs at the exposure time, and were also not suitable for the impermeability cryopreservation system.

Hydrogel is a novel soft material and has been paid much attention because of its unique properties, such as its excellent biocompatibility to the living body and biodegradation, and so it has been extensively applied in tissue engineering, clinical medicine, and drug delivery. In cell encapsulation, hydrogel capsules have a unique three-dimensional network structure, which allows the small molecules (e.g., nutrients and metabolic waste) to pass through the membrane without influencing the normal functions of cells and also provide immune protection against foreign substances [27]. In addition, the related investigations show that the hydrogel capsules are beneficial to minimize cell damage during the cryopreservation process [28,29,30], which can confine the ice crystal growth in its three-dimensional network, and keep the minor change rates of osmotic shock when the CPA permeates into cells. Hence, living cell encapsulation in hydrogel has recently become a simple, safe, and dependable method and is employed in the field of cryopreservation as a powerful supplementation to the traditional protocols of the storage of living cells. With regard to the cryopreservation of encapsulated cells, major studies have focused on the alginate and chitosan hydrogels, which were capable for the cryopreservation of a variety of cell types, while the current research regarding novel hydrogel capsules and supramolecular gels promotes the further development of the cryopreservation of living cells, which has attracted a great deal of interest from researchers in related fields [6].

In this paper, different hydrogel cryopreservation systems, including natural polymer hydrogel, synthetic polymer hydrogel, and supramolecular hydrogel systems, were summarized, and the role of these hydrogel cryopreservation systems in understanding protection mechanisms was also discussed.

## 2. Natural Polymer Hydrogel Cryopreservation System

### 2.1. Alginate Hydrogel Cryopreservation System

Alginate is a kind of natural polysaccharide extracted from seaweed and is a commonly used material for cell encapsulation because of its abundance and good biocompatibility. For cell cryopreservation based on microencapsulation technology, a variety of alginate capsules demonstrated a high efficiency in encapsulating living cells, aggregation and tissues, which could optimize the cryopreservation procedure, improve the survival rate and retain the normal functions of cells more than the traditional methods [31,32,33].

The alginate hydrogels are obtained by the crosslinking between alginate and some divalent or trivalent cations. The crosslink can be achieved at room temperature, and the common cations, Ca^2+^, Ba^2+^ and Sr^2+^ are dedicated to forming biocompatible alginate hydrogels for biomedical and biological applications [34,35]. In the field of the cryopreservation of living cells, as mentioned above, the formation of ice crystals and the use of high-concentration CPAs lead to the loss of cell viability and function, and so it is necessary to reduce the addition of CPAs and synchronously decrease the amounts of ice crystals during the cooling and thawing steps. Compared with standard slow freezing or vitrification methods, the ice crystal growth was inhibited in a three-dimensional network of hydrogel, but not open as with the former, and the osmotic pressure was also minimized when CPAs permeated into cells, which guaranteed a low CPA as well as ice crystal formation and osmotic injury. With respect to the storage of living cells in hydrogels, the existing studies have indicated that alginate hydrogels can effectively reduce the dosage of CPAs and minimize the amounts of ice crystals for high levels of viability and the maintenance of normal function.

**Figure 1:**
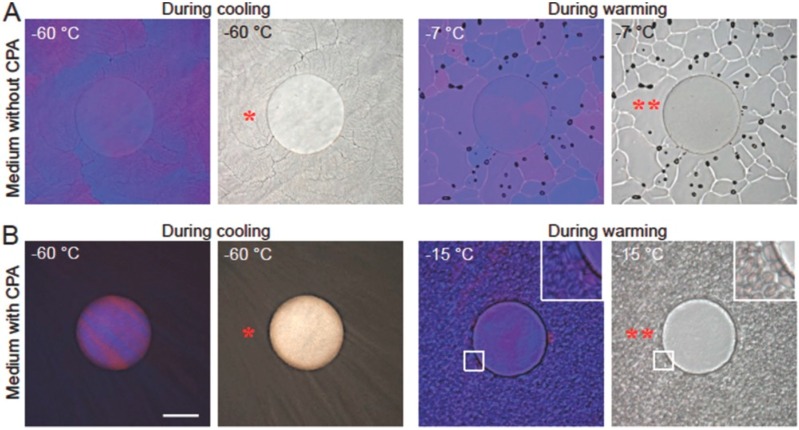
The intracellular ice was hindered in an alginate hydrogel microencapsulation [36]. (**A**) without CPA; (**B**) with CPA; CPA: 1.5 mol/L 1,2-propanediol and 0.5 mol/L trehalose; Color image: polarized; grayscale image: phase contrast; Scale bar: 100 μm.

Huang and co-workers reported a low-CPA vitrification method to cryopreserve mouse embryonic stem cells and human adipose-derived stem cells in a conventional plastic straw [36]. After cells were encapsulated in alginate hydrogel via a nonplanar microfluidic device, the cells microencapsulated in alginate hydrogel had an obvious impact on inhibiting intracellular ice formation when cells were thawed at physiological temperature (Figure 1). The ice formation inside the microcapsule was inhibited throughout the whole thawed process. Compared with the traditional low-CPA vitrification using a quartz microcapillary, the alginate hydrogel cryopreservation system achieved a low-CPA concentration of 2 mol/L (approximately four times lower) and a sample volume of 250 µL (100 times larger). Obviously, the preferential vitrification of the bulk solution occurred outside the alginate microcapsules, which inhibited the ice crystal injuries of cells in alginate hydrogel microcapsules due to the devitrification during warming. The alginate hydrogel cryopreservation system can effectively confine the ice formation more than the common slow freezing method.

Compared with cells, tissues have more complex structures and inner interaction systems including the compact stroma tissue and cell types, and so their encapsulation and cryopreservation must be distinguished. Xu and co-workers investigated in follicle maturation (IFM) using a slow-freezing strategy in an alginate hydrogel cryopreservation system, which was an option for the fertility maintenance of special human [29]. During the freeze–thaw process, partial follicles survived and grew into a mature oocyte. In this regard, the authors cryopreserved the ovarian tissue in both cortical strips and stroma in follicles (Cryo-Ov) and individually isolated follicles (Cryo-In). Subsequently, the three groups, Cryo-Ov, Cryo-In and non-cryopreserved control follicles, were cultured in an alginate hydrogel system with a unique three-dimensional structure for 12 days, which served as an assessment of follicle growth and oocyte maturation. The results of a culture period of 12 days showed that the Cryo-Ov group had lower androstenedione levels and the Cryo-Ov and Cryo-In group had a higher ratio of progesterone to estradiol. At the culture stage of 6 days, the cryopreservation group had a decreased Gja1 (Gap junction protein alpha 1, known as connexin 43) and Gja4 (Gap junction protein alpha 4 known as connexin 37) mRNA expression, and the Gja1 mRNA expression of three groups arrived at the same level after 12 days. Thus, the thawed follicles could maintain good viability and growth characteristics as fresh follicles, and could also be matured in eggs. The alginate hydrogel maintained follicular cell–cell and cell–matrix connections by the structure of a three-dimensional matrix, which was important for proper follicle development and oocyte maturation. Thus, the alginate hydrogel cryopreservation system can be applied in the cryopreservation of more complex cells or tissues.

In Perteghella’s work, buffalo (*Bubalus bubalis*) spermatozoa and Holstein Friesian (*Bos taurus*) semen were encapsulated and cryopreserved [37], which served for the monitoring of buffalo’s ovarian activity affected by climate change. Besides this, the defects of artificial insemination (AI) were also covered. The basic freezing and thawing process included an equilibration of 3 h at 4 °C, with the sustained spermatozoa freezing steps being from +4 °C to −10 °C (rate: −5 °C/min in bovine, −3 °C/min in buffalo), −10 °C to −100 °C (rate: −30 °C/min in bovine, −40 °C/min in buffalo), and −100 °C to −140 °C (rate, −20 °C/min in bovine, −20 °C/min in buffalo) and subsequent thawing at 37 °C for 1 min. The results showed that the progressive motility, path average velocity and pregnancy rates in either species were not obviously decreased, and the detrimental effects in the encapsulation process had not been observed.

In the actual cell-based application, the survival and function of cells in short-storage could be retained effectively in alginate capsules. Chen and co-workers used alginate hydrogels to encapsulate human mesenchymal stem cells (hMSCs) and mouse embryonic stem cells (mESCs) for short-term storage of stem cells [38]. After successful storage within alginate hydrogels for 5 days at 18–22 °C in sealed cryovials, the cell viability of hMSC and mESCs released from hydrogels achieved 74% and 80%, respectively. Moreover, the proliferation and expression of routine cell markers of hMSCs and mESCs showed no significant difference between alginate-encapsulated cells and cells cryopreserved in liquid nitrogen. The microcapsule membrane could cryopreserve the common cells, but this also limited and hindered some special functions of partial cell specials such as the adherence, migration, proliferation and differentiation of adherent cells.

**Figure 2:**
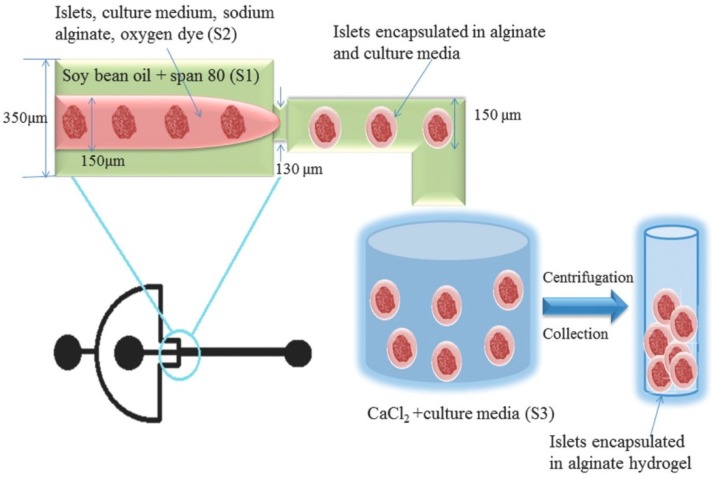
Encapsulation process of rat islets in microchannels [39].

**Figure 3:**
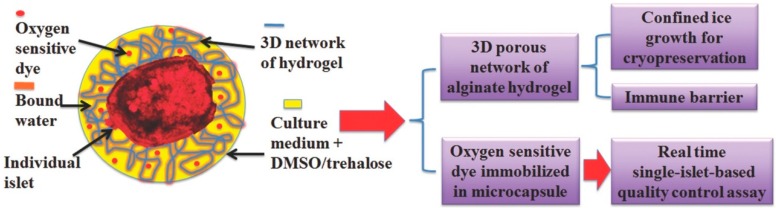
Quality control assay of cryopreserved pancreatic islets in microcapsules [39].

The encapsulation-based cryopreservation technique is an effective method to monitor the activity and assess the quality of living cells and obtain relevant numbers of characterized cells prior to cell-based therapy. For example, Chen and co-workers proposed a novel islet quality assays method. The single rat pancreatic islets and fluorescent oxygen-sensitive dye (FOSD) were encapsulated in alginate hydrogel microcapsules via microchannels (Figure 2) [39], and single-islet capsules embedding FOSD were cryopreserved for islet function simulation and evaluation. Here, FOSD was embedded to label and characterize the real-time oxygen uptake of individual islets. In the cryopreservation system of islet capsules with FOSD, the whole process of encapsulation and the cooling/thawing procedure had little impact on the function of FOSD, which provided a reliable assessment. Correspondingly, adenosine triphosphate (ATP), static insulin release measurement, and the oxygen consumption rate were tested for the assessment of the functions of encapsulated single-islet microcapsules after storing for 1 days and 7 days at −80 °C (Figure 3). These results implied that an individual-islet-based quality control method was proved simply and reliably for the fast and real-time assessment of the islet processing procedure in transplantation. A further result revealed that the alginate capsules benefitted the increased survival rate of thawed islets and the islets could release insulin in the alginate capsules. In addition, trehalose, added as a cryoprotectant during the freezing process, was suitable to improve the activity of the thawed islets. It is notable that the dye acted as a real-time single-islet oxygen sensor, which could analyze the viability and function of islets. This method offered a simple and real-time strategy to assess the viability of a single islet and enriched available islet source and also had a potential to face the current challenge in islet transplantion.

**Figure 4:**
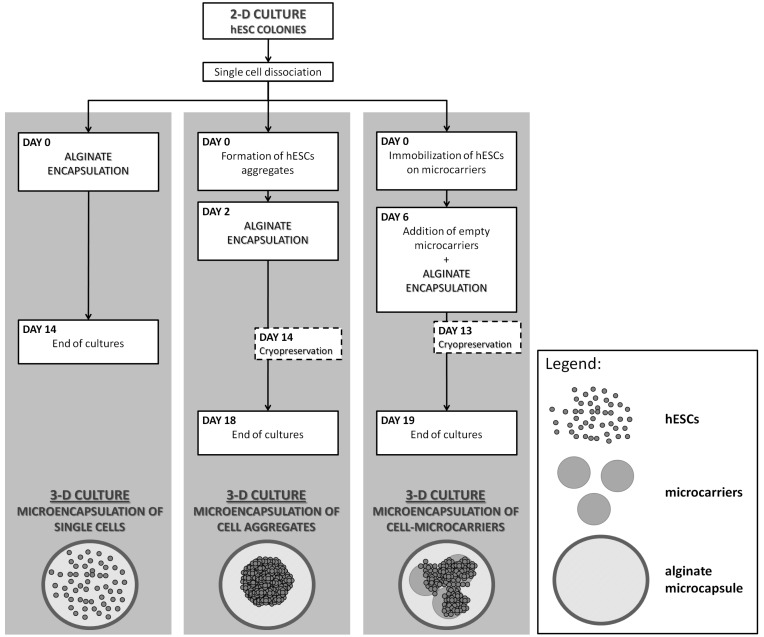
Three microencapsulated 3D culture strategies [40].

In clinical and industrial applications, the production of human embryonic stem cells (hESCs) with the feature of cell integrality and their long-term storage is a major challenge. An integrated expansion and cryopreservation strategy for hESCs was reported by Serra and co-workers [40]. Single cells, aggregates and immobilized microcarriers were microencapsulated (Figure 4). The microcarriers and aggregates were obtained after culturation for 13 and 14 days, and were pre-treated with 5 mM ROCKi (Rho kinase (ROCK) inhibitor) for 1 h for the following cryopreservation. In the freezing process, the cryopreservation equilibration was conducted for 20 min at 4 °C and subsequently frozen to −80 °C at a rate of 1 °C/min. The thawing process was conducted quickly on cryovials in a 37 °C water bath, diluted and cultured in certain conditions for a further assessment. The results showed that the hESCs lost viability quickly in single cell microcapsules and differentiated spontaneously in aggregate microcapsules. Furthermore, the results of the microencapsulation of hESC microcarriers showed that cell concentrations increased approximately twice and exhibited over 70% of cell recovery yield, and three times the survival rate of encapsulated hESCs compared with non-encapsulated cells, which was a prospective protocol for the scalable production and storage of pluripotent hESCs in a synergetic bioprocess. The microcapsulation of stem cells was a benefit to the culture of its aggregates, which could adjust the size of aggregates and maintain the integrality properties of cells for more than 2 weeks. As opposed to the traditional three-dimensional cell culture system, this research was promising to apply to clinical medicine, and combined cell proliferation with cell cryopreservation.

### 2.2. Chitosan/Alginate Hydrogel Cryopreservation System

The permeability and morphology of alginate capsules, and the biocompatibility of membrane materials, are related to cell cryopreservation. Thus, some biocompatible compounds, such as poly-l-lysine [41,42,43], chitosan [44,45,46], amino acid and derivatives [47,48], gelatin [49,50] and collagen [51] are usually employed for the chemical modification of the alginate capsule membrane. Based on different cell encapsulation requirements, recently, these modified alginate hydrogels have been gradually designed for the protection of various cells in the corresponding cryopreservation systems. For the high survival rate of probiotic bacteria *S. phocae* PI80, the frozen parameters were optimized with different cryopreservation methods and cryoprotectants by Kanmani and co-workers [52]. In the common cryopreservation method, the relative viability of *S. phocae* PI80 was retained at 74.6 ± 5.9% in an optimal trehalose system at −20 °C after storage for 6 months. When *S. phocae* PI80 cells were encapsulated with alginate–chitosan hydrogels, a high survival rate of *S. phocae* cells with high bacteriocin activity was obtained at −20 °C after storage for 6 months. Furthermore, the immobilization of cells was in a position to resist an acidic environment in simulated gastrointestinal conditions. The capsules were broken after 6 h in vivo treatment, and probiotic cells could enter the intestinal tract.

The results of Hardikar and his co-workers also showed that islets could perform with higher viability and functionality than the routine method in the chitosan–alginate encapsulation system [53]. The islet was encapsulated and cryopreserved via a routine method. Here, an islet-suspended solution in sodium alginate at the ratio of 500 islets/mL to alginate was used for the formation of islet beads in a microfluidic method. The cryopreservation was conducted including equilibration for 5 min at 22 °C in a 0.1 mL of 2 mol/L dimethyl sulfoxide (Me2SO), 0.4 mL of 3 mol/L Me2SO for 25 min at 0 °C, and finally, 2 mol/L Me2SO for 5 min at −7.5 °C. The thawing process was at 37 °C in a water bath of cryovials. The authors indicated that the islets used trypan blue (0.4% *w*/*v*, 0.4 g/100 mL solution) for the assessment of the percentage of islet viability. Different groups including encapsulated, nonencapsulated and freshly isolated groups were compared, and the results showed that the cell viability of the encapsulated group (value: 95.4 ± 1.3%) was much higher than that of the nonencapsulated group (value: 69.4 ± 3.5%), which maintained the same level as that of the freshly isolated group (value: 97.5 ± 0.8%). Insulin release in glucose at a concentration of 16 mmol/L revealed a similar effect, and the value of the encapsulated, nonencapsulated and freshly isolated groups were 247 ± 14 mU/10 islets, 110 ± 32 mU/10 islets and 255 ± 27 mU/10 islets, respectively.

To date, the development of alginate-based microencapsulation has been demonstrated to optimize freezing and thawing procedures and minimize cryo-injury; however, there are still some problems in developing cryopreservation protocols for microencapsulated cells. It is difficult for he alginate capsules used for cell protection to match the size of the encapsulated cell and simultaneously ensure specific biocompatibility [42,47,54], which gives an opportunity for the extra space for ice formation to bring about ice crystal injury. Besides this, the poor uniformity and fragile semipermeable alginate membrane reduce the chance of CPAs entering the capsules, which causes a low efficiency of CPAs. During the freezing process, unpredictable thermal stress may induce the formation of cracks in the weaknesses of the capsule material, which dramatically changes the capsule permeability for the cell damage. The difficult removal of the capsule membrane also limits its application, and so a novel alginate modified method may be beneficial to extend the alginate-based hydrogel cryopreservation system.

## 3. Synthetic Polymer Hydrogel Cryopreservation System

Compared with natural polymer hydrogel, synthetic polymer hydrogel has tunability and designability of its matrix crosslinking by using different reaction mechanisms. The structure and performance of synthetic polymer hydrogel may be adjusted to the required permeability in cell encapsulation and cell species exchange [55].

**Figure 5:**
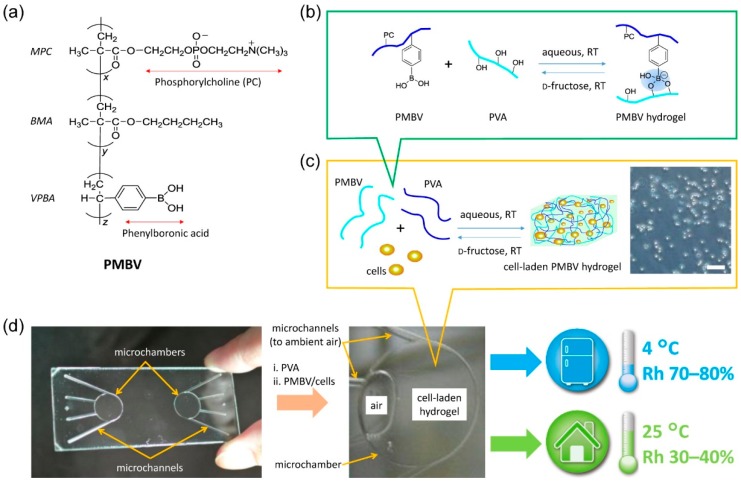
Spontaneous packaging and hypothermic storage of cells in stoichiometric conditions [56]. (**a**) PMBV hydrogel; (**b**,**c**) cross-linking reaction between PMBV and PVA; (**d**) microchanels in a glass microchip. Scale bar is 100 μm (**c**). PMBV: poly (2-methacryloyloxyethyl phosphorylcholine-*co*-*n*-butyl methacrylate-*co*-*p*-vinylphenylboronic acid); MPC: methacryloyloxyethyl phosphorylcholine; BMA: *n*-butyl methacrylate; VPBA: *p*-vinylphenylboronic acid; PVA: poly (vinyl alcohol).

Bionic simulated cell culture and cryopreservation in laboratories are very important for understanding the interaction mechanism of the cell system. Xu and co-workers presented a spontaneous packaging and hypothermic storage technology by a cell-membrane-mimetic methacryloyloxyethyl phosphorylcholine (MPC) polymer hydrogel incorporated within a glass microchip (Figure 5) [56]. The results showed that the MPC polymer hydrogel in microchannels was capable of maintaining the cell viability and intracellular esterase function, cell membrane integrity and morphology. Subsequently, except for a tiny morphology change, the above indicators were retained for more than 1 week at 4 °C and at least 4 days at 25 °C, and the whole process was easy to achieve in existing laboratories.

For the freeze–thaw process, the state transition of a cell cryopreservation system such as the crystallization of water produced polymer–solvent phase separation, which offered an opportunity for non-covalent bond hydrogel formation. Vrana et al. encapsulated bovine arterial smooth muscle cells within PVA (poly (vinyl alcohol))–gelatin hydrogels [57]. The effects of serum presence, DMSO concentration, coagulation bath, and PVA viscosity on cell viability and gel stability were investigated. The concentrations of DMSO and serum were beneficial for high cell viability, and the gelation process could not affect the cell viability. Attributable to the designability of the polymer in the structure, the synthetic polymer hydrogel can store the cells in a polymer matrix for a tissue-engineering application and further adjust its expected properties, which was superior to natural polymer hydrogel.

Minkle Jain and colleagues synthesized a dextran-based polyampholyte hydrogel which was suitable for the encapsulation cryopreservation of murine fibroblasts [58]. The structure of this hydrogel was derived from the amination of Poly-l-lysine and azide groups in dextran, and the carboxylation of partial amino groups. The dextran-based polyampholytes showed good cryoprotective properties, which could form in situ hydrogels with desired amounts of dibenzylcyclooctyne dextran (DBCO-Dex) via Cu-free click chemistry. The cryoprotective properties depended on the polymer concentration, the number of amino groups introduced into the dextran, the ratio of carboxylation, and the osmolarity of the solution. The survival rate of L929 cells encapsulated in the dextran-based polyampholyte hydrogels without any additional cryoprotectants was over 90% after thawing, and the result indicated that these hydrogels served as a CPA, and also have the ability to protect cells from freezing damage but without high cytotoxicity. Thus, these hydrogels appear appropriate to serve as scaffolds for the field of tissue engineering due to their excellent cryoprotective properties and high biocompatibility.

## 4. Supramolecular Hydrogel Cryopreservation System

Supramolecular gel is a physically crosslinked gel which is self-assembled by gelators via a non-covalent bond reaction in water and organic solvent medium [59]. The gelator stems from the organic compounds, which gels in water or organic solvent, and extends to cholesterol [60], amino acids and their derivatives [61], polypeptides [62], metallic organic compounds [63], sugars and their derivatives [64]. The weak force of interaction [65], such as hydrogen bonds [66,67], van der Waals forces [68], hydrophobic interactions [69,70], as well as π-π interactions [71], is analogous to the biological mechanism of weak reaction, which shows good compatibility. The reversible property and low activation energy of the non-covalent bond has potential in stimuli-responsive and self-repairing functions. In the case of water being used as a medium, supramolecular hydrogel has the main driving force of hydrogen bond.

**Figure 6:**
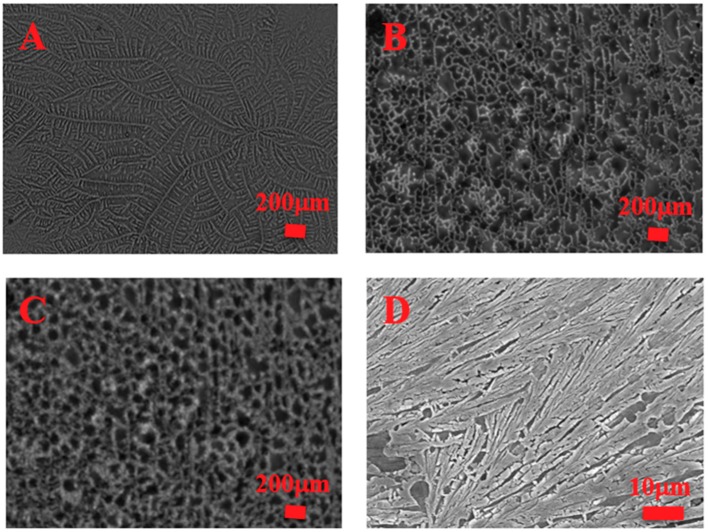
Morphology images of BDTC (Boc-*O*-dodecyl-l-tyrosine cultured) supramolecular gel in different conditions [6]. (**A**) 4 °C; (**B**) −20 °C; (**C**) −80 °C; (**D**) FE-SEM image of the BDTC gel.

Chen’s group reported a novel supramolecular gel cryopreservation system (BDTC gel system, Boc-*O*-dodecyl-l-tyrosine cultured supramolecular gel system) for neural cell cryopreservation [6]. The morphology of supramolecular gel at different temperatures and thermal analysis implied that tangled fiber structure of the supramolecular gel could confine the ice crystal growth and minimize the amount of ice crystals (Figure 6), and on the other hand, it decreased the freezing point of the BDTC gel system to −18.2 °C. Furthermore, PC12 cells and Schwann cells encapsulated in this supramolecular gel were cryopreserved at −80 °C for 7 days. Trypan blue exclusion, fluorescent staining and MTT assays were used for the assessment of the viability and proliferation of thawed PC12 cells and Schwann cells. Ascribed to the 3D porous dendritic network structure of the BDTC gel system in inhibiting ice crystal growth, the viability of thawed PC12 cells and Schwann cells in the BDTC gel system was 10.2% and 11.1% higher than in the groups without a gelator, respectively. In the thawing process, supramolecular gel culture medium was transferred into liquid via a routine thermoreversible phase transition process and was easily removed in the process of centrifuging and washing; thus, the adherence, spreading, and proliferation of adherent cells was as normal as the fresh cell, which was entirely distinct from that of the above alginate hydrogel cryopreservation system.

**Figure 7:**
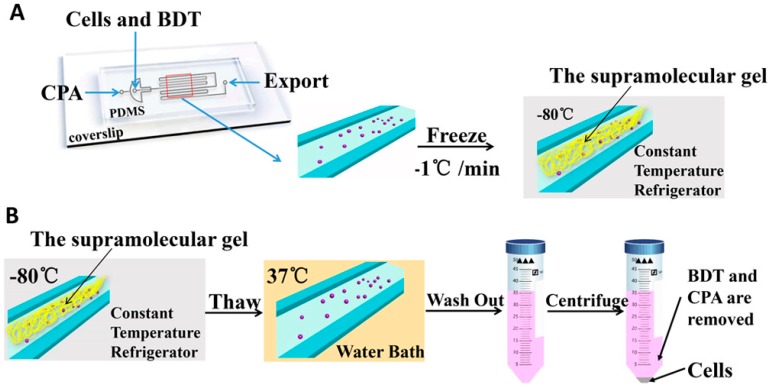
Process of cell freeze–thawing in the microchannel [72]. BDT: Boc-*O*-dodecyl-*L*-tyrosine; PDMS: polydimethylsiloxane. (**A**) cell cryopreservation; (**B**) cell thawing.

Chen’s group also investigated a novel supramolecular hydrogel cryopreservation system in a microchannel (Figure 7) [72]. The gelator Boc-*O*-dodecyl-l-tyrosine (BDT) self-assembled successfully to a supramolecular gel (BDTC) in microchannels for cell cryopreservation. Due to the designability of the microchannel, the microstructure of supramolecular hydrogel could be adjusted to the shape and size of microchannel architecture, and an effective cell cryopreservation system in the microchannel could be built. The authors introduced a specially designed microchannel for the self-assembly of the BDT gelator into BDTC, which was confined into a more compact three-dimensional network structure than the aforesaid research without microchannels. This compact structure inhibited the formation of reduced ice crystals, decreased the change rate of cell volumes and osmotic shock and the freezing point of the cryopreservation system at higher levels, and so a higher viability was obtained than the cells which were cryopreserved in cryovials. The growth and proliferating functions of thawed RSC96 cells extruded from the microchannels were similar to the fresh cells. The supramolecular gel microstructure could be adjusted in the microchannel. Attributable to the desired shape and size of the microchannel, the supramolecular hydrogel cryopreservation system via the microchannel has a wider range than the individual other hydrogel cryopreservation systems, sand o it would be expected to be used in cell therapy, tissue engineering and organ transplantation.

## 5. Materials and Methods

Different hydrogel cryopreservation systems are listed in Table 1.

## 6. Conclusions

The cell cryopreservation is the main method for the long-term storage of living cells in cell-based applications. For the natural polymer hydrogel cryopreservation system, alginate or chitosan hydrogel encapsulated the initial cell via a permeable protective mechanism, which was cryopreserved at different conditions; thus, the ice formation was confined in the porous three-dimensional network structure, which minimized the cell damage. The efficiency of this cryopreservation technique depended on the shape, morphology, and size of microcapsules and the intrinsic properties of the microcapsule membrane. The synthetic polymer hydrogel has a good flexibility in its structure and properties for cell cryopreservation. However, the hydrogel membrane material with a strong chemical bond reaction could be difficult to remove after cell thawing, which could have an impact on the viability and function of thawed cells. For the supramolecular hydrogel, its weak reaction was suitable for encapsulation and cryopreservation and being removed after cell thawing. However, the different category of the supramolecular hydrogel is yet to be developed and the cell types which are cryopreserved should also be extended. The association of other technologies, such as multicomponent CPA cryopreservation and the microchannel method, may be the subject of future research in hydrogel cryopreservation systems.

## Figures and Tables

**Table 1:** Articles reporting on natural polymer hydrogel, synthetic polymer hydrogel, and supramolecular hydrogel cryopreservation systems.

Citation	Hydrogel Cryopreservation System	Main Materials	Cyropreservation	Viability
Huang et al., 2015 [36]	Alginate	mESCs/hADSCs; PROH; trehalose	Freezing: −60 °C; Thawing: ~24 °C; Rate: both 60 °C/min	Most of the microencapsulated cells could survive
Xu et al., 2009 [29]	Alginate	Ovaries ^1^; Follicles; Sucrose; DMSO	Freezing: (1) 4 ~ −9 °C at −2 °C/min; (2) 6 min at −9 °C; (3) seeded manually for 4 min at −9 °C; (4) −9 ~ −40 °C at −0.3 °C/min; Thawing: ~22 °C for 30 s; plunged into a 37 °C water bath with gentle shaking	Fresh-In: 78.0 ±7.7% ^2^; Cryo-Ov: 71.7 ± 10.9%; Cryo-In: 73.7 ±13.8%
Perteghella et al., 2017 [37]	Alginate	May–July: Mediterranean Italian water buffalo (*B. bubalis*); Holstein Friesian bulls (*Bos taurus*); BULLXcell extender solution	Freezing: +4 °C~ −10 °C (rate: −5 °C/min in bovine, −3 °C/min in buffalo), −10 °C ~ −100 °C (rate: −30 °C/min in bovine, −40 °C/min in buffalo), and −100 °C ~ −140 °C (rate, −20 °C/min in bovine, −20 °C/min in buffalo); Thawing: 37 °C for 1 min	No relative test
Chen et al., 2013 [38]	Alginate	Mouse ESCs;DMEMIsopropyl alcohol	Mouse ESCs and hMSCs: stored at room temperature (18 °C ~ 22 °C, atmospheric CO_2_); ~ −80 °C for overnight (rate: 1 °C/min); Thawing: incubation for 4 min in the alginate-dissolving buffer	Mouse ESCs: 74%; hMSCs: 80%
Chen et al., 2016 [39]	Alginate	Islets; oxygen-sensitive dye Pt (II) meso-tetra (N-methyl-4-pyridyl) porphine tetrachloride; DMSO; Trehalose	Freezing: 4 °C ~ −80 °C (rate: −1 °C/min); Thawing: quickly thawed in water bath (37 °C)	No relative test
Serra et al., 2011 [40]	Alginate	hESCs; DMEM; Isopropanol; DMSO	Freezing: 4 °C ~ −80 °C (rate: −1 °C/min); Thawing: quickly thawed in water bath (37 °C)	Over 70%
Kanmani et al., 2011 [52]	Chitosan/Alginate	probiotic bacterium *S. phocae* PI80; glucose, sucrose, trehalose, galactose, glycerol	Freezing: room temperature ~ −20 °C; −20 °C dried under vacuum for 20 h; stored at −20, 4, 25, and 35 °C for six months; Thawing: thawed at room temperature for 1 h	*S. phocae* PI80: 74.6 ± 5.9% after stored at −20 °C for six months
Hardikar et al., 2000 [53]	Chitosan/Alginate	Islets; DMSO	Freezing: 22 °C ~ 0 °C for 25 min; 0 °C ~ −7.5 °C for 5 min; Thawing: quickly thawed in water bath (37 °C)	Encapsulated islets: 95.4 ± 1.3%
Xu et al., 2015 [56]	Synthetic polymer	L929 cells; DMEM; PMBV	Freezing: room temperature ~ 4 °C; Thawing: quickly thawed in room temperature	No relative test
Vrana et al., 2009 [57]	Synthetic polymer	bovine thoracic arterial smooth muscle cells; PVA	Freezing: 4 °C for 1 h; −70 °C overnight; Thawing: thawed in water bath (37 °C) for 10 min; prewarmed serum was added to remove DMSO every 15 min	50% (affected by the concentration of the serum and DMSO)
Jain et al., 2014 [58]	Synthetic polymer	L929 cells; DMEM; dextran-based polyampholyte; DMSO	Freezing: −80 °C overnight; Thawing: quickly thawed	[4:1(mass ratio) azide-Dex-PA(0.69): DBCO-Dex]: 93 ± 4.2%
Zeng et al., 2016 [6]	Supramolecular	Boc-l-tyrosine methyl ester; 1-bromododecane; PC12 cells; Schwann cells; DMSO	Freezing: 4 ~ −80 °C (rate: −1 °C/min); 0 °C ~ −7.5 °C for 5 min; Thawing: quickly thawed in water bath (37 °C)	PC12 cells: 10.2%; Schwann cells: 11.1%
Lan et al., 2018 [72]	Supramolecular	Boc-l-tyrosine methyl ester; 1-bromododecane; RSC96 cells; DMSO; Ethylene glycol; Trehalose	Freezing: 4 °C ~ −80 °C (rate: −1 °C/min); 0 °C ~ −7.5 °C for 5 min; Thawing: quickly thawed in water bath (37 °C)	62.5% ~ 83.9% in different cryoprotectants

mESCs = murine embryonic stem cells; hADSCs = human adipose-derived stem cells; DMSO: dimethyl sulfoxide; PROH = 1,2-propanediol; DMEM = Dulbecco’s modified Eagle’s medium; hMSCs = Human mesenchymal stem cells; hESCs = Human embryonic stem cells; PMBV = a 2-methacryloyloxyethyl phosphorylcholine (MPC) polymer-based hydrogel. PVA: poly(vinyl alcohol). ^1^ removed from prepubertal, 12-day-old female F1 hybrids (C57BL/6j×CBA/Ca); ^2^ Values are the average ±SD of multiple follicles from three or four independent cultures.
